# Supplementary material for: In Staphylococcus aureus, MbcS is a refunctionalized acyl-CoA synthetase that confers a fitness advantage during intra-species competition
Source: J Bacteriol. 2026 Aug 27;208(9):e00185-26. doi: 10.1128/jb.00185-26 (PMC13584741; doi:10.1128/jb.00185-26)
Supplement: Supplementary file 1 [file jb.00185-26-s0001.docx]

**Supplemental material**

**Figures and legends**

**Figure S1. Phylogenetic analysis suggests that presence of *mbcS* in human-associated staphylococci is a result of a refunctionalization event.** The *Staphylococcus* and outgroup species used in the phylogenetic analysis are depicted in the second innermost ring. The three major protein families formed from all acyl-CoA synthetases, and AMP-binding proteins are depicted as the blue, green, and yellow outer lines. All MbcS-like proteins (outer ring, red) clustered together in the protein family 2 (green outer line).

**Figure S2. SDS-PAGE gel from purified Ptb and Buk from *S. pseudintermedius*.** The proteins phosphotransbutyrylase Ptb (32.7 kDa) and butyrate kinase Buk (39.4 kDa) from *S. pseudintermedius*, both fused to a TEV-cleavable *N*-terminal hexahistidine (His_6_) tag (1.71 kDa), were purified to near apparent homogeneity as described in *Materials and Methods*. PM, protein marker.

**Figure S3**. **Inducing *Spptb*^+^ and *Spbuk*^+^ expression does not increase growth of *lpdA::kan^+^ mbcS::erm^+^* double mutant in CDM supplemented with *i*C_4_.** WT cells with pWY53 (pCT3 empty vector; black bar) and *lpdA::kan^+^ mbcS::erm^+^* double mutant cells with pWY53 (grey bar), pWY54 (pCT3 with *SambcS*^+^; green bar), or pMF020 (pCT3 with S*pptb*^+^-*buk*^+^; orange bar) were grown in CDM only or CDM complemented with either (**D**) 1 mM or (**E**) 10 mM of the respective BCCAs, with 10 ng ml^-1^ anhydrotetracycline as inducer. Optical density at 600 nm (OD_600_) was measured after 20 h and 40 h (data not shown). Data are plotted as mean ± SD from three biological replicates. ******p<0.0001, ***p<0.001, **p<0.01, *p<0.05 one-way ANOVA with Tukey’s multiple comparison test within each condition; ns, not significant.

**Figure S4. Putative repressors of *mbcS* transcription*.*** Plasmid pAP4 containing the *mbcS^+^* promoter region fused translationally to *gfp* (P*_mbcS_-gfp*) was simultaneously transduced into 111 transcription factor mutant strains of *S. aureus* distributed across two 96-well plates (**A**) and (**B**). Cells were grown in chemically defined medium supplemented with 10 g L^-1^ tryptone overnight with shaking. Cells were then pelleted, washed twice with PBS, and promoter activity was measured (relative fluorescence units [RFUs]; GFP/OD_600_). WT strain with pAP4 was used as the baseline control. The putative repressors of *mbcS* are depicted with the colored bars. ****p<0.0001, *p<0.05, one-way ANOVA with Dunnett’s multiple comparison test relative to WT; n=3.

**Figure S5*.* Wild-type cells out-compete the *mbcS::erm^+^* mutant during growth in rich, complex medium**. (**A**) Initial CFU counts (input) from monocultures started with 10^5^ of WT cells carrying pKK22 empty vector (Tm^R^, WT 1 + EV) or pCT3 empty vector (Tet^R^, WT 2 + EV), and *mbcS::erm^+^* cells carrying pCT3 empty vector or pWY54 (pCT3 with pS*ambcS*^+^) (marked with Em^R^ and Tc^R^) in 1 ml of plain TSB. (**B**) Initial monocultures were incubated overnight at 37^o^C and final CFUs were enumerated (output). (**C**) Initial and (**D**) Final CFU counts from co-cultures were performed as described in *Materials and Methods*. The vertical dashed lines delineate the independent co-cultures; open and closed shapes denote the strains present in the independent co-cultures. Data are plotted as mean ± SD from six biological replicates. **p<0.01, one-way ANOVA with Tukey’s multiple comparison test for panels **A**, **C**, and **D**; Kruskal-Wallis test with Dunn’s multiple comparison test for panel **B**. ns, not significant.

**Tables**

**Table S1. Strains used in this study.**

| Strain | Genotype or description | Source or reference ^†^ |
| --- | --- | --- |
| BL21 (DE3) | *E. coli* strain harboring a T7 expression system; for protein overexpression | (1) |
| DH5α | F^–^ φ80*lac*ZΔM15 Δ(*lac*ZYA-*arg*F) U169 *rec*A1 *end*A1 *hsd*R17(r_K_^–^, m_K_^+^) *pho*A *sup*E44 λ^–^*thi*-1 *gyr*A96 *rel*A1 | New England BioLabs |
| RN4220 | Restriction-deficient, highly transformable *S. aureus* strain | (2) |
| SRB687 | USA300 LAC CA-MRSA Em^S^ (AH1263) ("LAC") | (3) |
| SRB892 | LAC *codY::*φΝΣ*^a^* [*erm::tet(M)*] | (4) |
| SRB1710 | LAC / pKK22 | (5) |
| SRB1712 | LAC *lpdA:*:φNΣ *mbcS1* (promoter up mutation in *mbcS*) | (6) |
| SRB2440 | LAC / pAP4 | (7) |
| SRB2889 | LAC *mbcS*::φNΣ |  |
| SRB3105 | LAC *lpdA::kan^+^* *mbcS::*φNΣ / pWY54 | (7) |
| SRB3107 | LAC *mbcS*::φNΣ / pWY53 | (7) |
| SRB3108 | LAC *mbcS*::φNΣ / pWY54 |  |
| SRB3143 | LAC / pWY53 | (7) |
| SRB3912 | *S. pseudintermedius* (clinical isolate) | Dr. Jessica Gilbertie |
| SRB4012 | LAC *lpdA::kan^+^* *mbcS::*φNΣ / pWY53 |  |
| SRB4019 | DH5α / pMF017 |  |
| SRB4020 | DH5α / pMF018 |  |
| SRB4022 | LAC *codY*::φNΣ [*erm::tet(M)*] / pAP4 |  |
| SRB4031 | LAC *codY*::φNΣ [*erm::tet(M)*] / pAP4 pCS02 |  |
| SRB4101 | DH5α / pMF020 |  |
| SRB4152 | RN4220 / pMF020 |  |
| SRB4153 | DH5α / pMF022 |  |
| SRB4154 | RN4220 / pMF022 |  |
| SRB4155 | LAC *lpdA::kan^+^* *mbcS::*φNΣ / pMF020 |  |
| SRB4156 | LAC *lpdA::kan^+^* *mbcS::*φNΣ / pMF022 |  |
| SRB4231 | LAC *srrA*::φNΣ |  |
| SRB4232 | LAC *srrA*::φNΣ / pAP4 |  |
| SRB4313 | LAC / pKK22 pAP4 |  |
| SRB4314 | LAC / pKK22 pMRSI |  |
| SRB4315 | LAC *codY*::φNΣ [*erm::tet(M)*] / pKK22 pAP4 |  |

^†^Unless otherwise indicated, the strains were constructed during the course of this study.

*^a^*φNΣ refers to NTML transposon mutant alleles containing the *erm^+^* allele, conferring Em^R^. For clarity throughout the manuscript, these mutants are designated xxx::*erm^+^.*

**Table S2. Oligonucleotides used in this study.**

| Oligonucleotides | Sequence (5’ 🡪 3’) | Usage |
| --- | --- | --- |
| oKM113 | TTTGGATCCTTATTTACTTTTTTCTAATTCATC | *codY^+^* reverse  primer containing  BamHI cutting site for cloning into pKK22 |
| oKM120 | AATTAAAGCGGCCGCTAATGACTTTCTAAATGGAAAATAC | *codY^+^* forward  primer containing  NotI cutting site for cloning into pKK22 |
| oMF202 | TGTATGAAAGGAAGTTAGTTTTGAATTTTACAGACTTGCTTAAAGAACCTCAAG | To amplify *Spptb^+^* for Gibson construction of pMF016 (forward fragment) |
| oMF203 | TGCGCCGAGAATAAGACTCGCAACTTTTGCACGACCAAAATATGTGAGTGATTTATATAA | To amplify *Spptb^+^*-FLAG for Gibson construction of pMF016 (reverse fragment) |
| oMF204 | GACAACAACACTCGTATTAAATTTAGGGAGTACCTCTAGTAAATATGCAATATATGAAAA | To amplify *Spbuk^+^* for Gibson construction of pMF016 (forward fragment) |
| oMF205 | TTTCTATTTGTCATCGTCATCCTTATAATCTTGATATATCTCAATTTTAATTTGCTGATT | To amplify *Spbuk^+^*-FLAG for Gibson construction of pMF016 (reverse fragment) |
| oMF206 | CAAGATTATAAGGATGACGATGACAAATAGAAATTCTTGAAGACGAAAGGGCCTCGTGAT | To linearize pWY54 for Gibson construction of pMF016 (forward fragment) |
| oMF207 | AGCAAGTCTGTAAAATTCAAAACTAACTTCCTTTCATACATCCACCGATATCA | To linearize pWY54 for Gibson construction of pMF016 (reverse fragment) |
| oMF208 | AAAACCTGTATTTTCAGGGCTTGAATTTTACAGACTTGCTTAAAGAACCTCAAG | To amplify *Spptb^+^* for Gibson construction of pMF017 (forward fragment) |
| oMF209 | GGCCCCAAGGGGTTATGCTATTAACCGACTTTTAATGCGACTAAAATAGAGTTG | To amplify *Spptb^+^* for Gibson construction of pMF017 (reverse fragment) |

**Table S2. Continued.**

| Oligonucleotides | Sequence (5’ 🡪 3’) | Usage |
| --- | --- | --- |
| oMF210 | TTAGTCGCATTAAAAGTCGGTTAATAGCATAACCCCTTGGGGCCTCTAAACGGG | To amplify pTEV5 for Gibson construction of pMF017 (forward fragment) |
| oMF211 | AGCAAGTCTGTAAAATTCAAGCCCTGAAAATACAGGTTTTCACTAGT | To amplify pTEV5 for Gibson construction of pMF017 (reverse fragment) |
| oMF212 | AAAACCTGTATTTTCAGGGCATGACAACAACACTCGTATTAAATTTAGGGAG | To amplify *Spbuk^+^* for Gibson construction of pMF018 (forward fragment) |
| oMF213 | GGCCCCAAGGGGTTATGCTACTATTGATATATCTCAATTTTAATTTGCTGATTC | To amplify *Spbuk^+^* for Gibson construction of pMF018 (reverse fragment) |
| oMF214 | AAATTGAGATATATCAATAGTAGCATAACCCCTTGGGGCCTCTAAACGGG | To amplify pTEV5 for Gibson construction of pMF018 (forward fragment) |
| oMF215 | AATACGAGTGTTGTTGTCATGCCCTGAAAATACAGGTTTTCACTAG | To amplify pTEV5 for Gibson construction of pMF018 (reverse fragment) |
| oMF217 | GCAAAAGTTGCGAGTCTTATTCTCGGCGCACATTTTCCAATCGTTCTTACATCAAGAGCGGACAGTATTGAAAATAAAATCAACTCTATTTTAGTCGCATTAAAAGTCGGTGATTATAAGGATGACGATGACAAATAATTTTGATTTGTTTTTGAAAGAAGGGATTGTATGACAACAACACTCGTATTAAATTTAGGGAG | gblock to generate *Spptb^+^*^-^FLAG (in pMF016) |

**Table S2. Continued.**

| Oligonucleotides | Sequence (5’ 🡪 3’) | Usage |
| --- | --- | --- |
| oMF222 | ATGAATAAACCCCATTTAGTGGCACCTGAAGATTTCAATATTGTCTCTGAGATTGAGAAGTACGCAAAAGATAAATCAAAAGTTGCGATCATCTTTGAAGATAATGCAGGTGGAACAACAGAAGTAACTTATGATGAATTAATTCGCAGCGCAAATCGCATTGGTAATTTATTTAAACAACATGGTTTGCGTAAAGGCGATACATTACTCATTAAAATGGAACGCAGTATTGAAACCTATGAAGTTTATATTGCGGCATTGAAATTAGGTGTCGCTTTAATCCCAGCATCTGAAATGTTAAGAACGAAAGACTTGCAGTACCGTATTACACACGGTGAAGTCGATGCAGTCTTATCTATTGCGTCAGGGGCAGATGAATTTGAAGGCGTAAAAGAATATGATGACTTAACAAAATTCATTATTGGGGATACTAAAGAAGATTGGATTAATGTCGAAGAAGGCAAAAAAGAAATGAGTGAGGAATTAGACATCACACCGACAAAACGTGATGACGTCGCTTTCTTACCTTATACATCAGGTACAACAGGCAATCCTAAAGCTGTTGTACACTCACATGGCTGGGGTTACGCACATATGCAGATGGCACCGAAACATTGGTTGAACATCAGTGAAGGTGACATTGTATGGGCCACTGCCGCACCTGGTTGGCAAAAATGGGTATGGAGTCCATTCCTATCTACAATGACATCCGGCGCAACAGCCTTTGTGTTTAATGGTCGCTTCAACGGCACAGTCTATTTAGAATTACTCGAAAAATACAAAATCAACGTACTTTGCTGTACGCCGACTGAGTACCGTATTATGGCCAAACTCCAAGATTTAGGTAGTTATGACCTATCTCATCTTCATGACGCTGTATCAGCCGGAGAACCTTTAAACCAAGAAGTTGTTGAGAAATTCCAAGATACTTTCGATATCACAGTACGTGACGGTTATGGTCAAACAGAAAGTACTTTAGTGATTGGTTTATTAAAAGATGTTAAAGGTAAACCAGGTTCTATGGGTAAAGCAATTCCTGGCAGTGAAGTCACTATTATTGACGATGAAGGTAACTTAGCTGAAGTCGGAGAAGTCGGCAATATCGCTGTTCCAGTCGATTTACCTGCATTGTTCAAAGGTTACTTCAAAGAGCCTGAACGTACACAAGAACGTATCGTCGGCAACTATTTCATGTCAGGCGACTTAGCTAAAGTTGATGAAGATGGCTACTTCTGGTTCGAAGGCCGTAAAGACGATATCATTATCAGCTCTGGTTACACAATCGGACCATTCGAAGTGGAAGATTCATTAACGAAACACCCTGCAGTTAAAGAATGTGCAGTGGTCGCAAGCCCACATGAATTACGTGGTAATATCGTAAAAGCCTTCATTATATTACAAGATGGCTATGAACCAAGTGATGACTTAGTGAAAGAACTTCAAAATTATGTCAAATACGATGTCGCACCTTATAAATATCCACGTGCGATTGAATTTGTTGAAGAATTACCAAAAACTAATTCAGGTAAAATCCGTCGTGTTGAATTACGTGAAGCAGAAATCGAAAAATACAATAAAAAATAA | gblock containing MUA81_01165^+^ coding sequencing from *S. simulans* IVB6244 |

| Oligonucleotides | | Sequence (5’ 🡪 3’) | Usage |
| --- | --- | --- | --- |
| oMF233 | AAAAATACAATAAAAAATAAAAATTCTTGAAGACGAAAGGGCCTCGTGATACGCC | | To linearize pWY54 for Gibson construction of pMF022 (forward fragment) |
| oMF234 | ACTAAATGGGGTTTATTCATAACTAACTTCCTTTCATACATCCACCGATATCAAG | | To linearize pWY54 for Gibson construction of pMF022 (reverse fragment) |
| oWY7 | CATCATTAATTCCTCCTTTTTGTTG | | To confirm fragments cloned into pCT3 (forward) |
| oWY33 | CCACCTGACGTCTAAGAAACC | | To confirm fragments cloned into pCT3 (reverse) |

**Table S2. Continued.**

**Table S3. Plasmids used in this study.**

| Plasmid | Description | Source or reference^†^ |
| --- | --- | --- |
| pMRSI | Promoterless GFP reporter vector, Cm^R^ | (8) |
| pTEV5 | Purification vector, N-terminal His_6_-tag, cleavable with rTEV protease, Ap^R^ | (9) |
| pCS02 | Stably maintained plasmid expressing *codY^+^* on native promoter, Tm^R^ |  |
| pKM25 | pKK30 with native promoter fused to *codY^+^* for complementation*,* Ap^R^*,* Tm^R^ | (10) |
| pKK22 | Stably maintained S. aureus complementation vector, Tm^R^ | (5) |
| pAP4 | pMRSI containing the *mbcS^+^* promoter region fused to *gfp*, Cm^R^ | (7) |
| pWY53 | *S. aureus* integration vector pCT3, Tc^R^ | (7) |
| pWY54 | pCT3 containing the *SAUSA300_2542* (*mbcS^+^*) gene with a C-terminus 3xFLAG sequence, Tc^R^ | (7) |
| pMF017 | *Sp*Ptb overproduction plasmid, Ap^R^ |  |
| pMF018 | *Sp*Buk overproduction plasmid, Ap^R^ |  |
| pMF020 | Codon optimized version of pMF016 without C-terminus FLAG-tag |  |
| pMF022 | pCT3 containing MUA81_01165 coding sequencing from *S. simulans* IVB6244 with *SambcS^+^* SD sequence, Tc^R^ |  |

^†^ Unless otherwise indicated, plasmids were constructed during the course of this study.

**Table S4. Multiple Reaction Monitoring (MRM) transitions**

| Metabolite/Mode | Parent Ion | Daughter Ion | Cone Voltage | Collision Energy |
| --- | --- | --- | --- | --- |
| CoA | 768.1 | 261.1 | 70 | 26 |
| IB-CoA | 838.1 | 331.1 | 16 | 32 |
| 3-HPMA-d3 | 225 | 163 | 15 | 15 |

**References**

1. STUDIER, F. W. AND B. A. MOFFATT (1986). "Use of bacteriophage T7 RNA polymerase to direct selective high-level expression of cloned genes." J Mol Biol **189**(1): 113-130.
2. KREISWIRTH, B. N., LÖFDAHL, S., BETLEY, M. J., O'REILLY, M., SCHLIEVERT, P. M., BERGDOLL, M. S. & NOVICK, R. P. (1983). The toxic shock syndrome exotoxin structural gene is not detectably transmitted by a prophage. *Nature.,* 305**,** 709-712.
3. BOLES, B. R., THOENDEL, M., ROTH, A. J. AND HORSWILL, A. R. 2010. Identification of genes involved in polysaccharide-independent *Staphylococcus aureus* biofilm formation. *PLoS One.,* 5**,** e10146.
4. MLYNEK, K. D., W. E. SAUSE, D. E. MOORMEIER, M. R. SADYKOV, K. R. HILL, V. J. TORRES, K. W. BAYLES AND S. R. BRINSMADE (2018). "Nutritional regulation of the Sae Two-Component System by CodY in *Staphylococcus aureus*." *Journal of Bacteriology* 200(8): JB.00012-00018.
5. KRUTE, C. N., KRAUSZ, K. L., MARKIEWICZ, M. A., JOYNER, J. A., POKHREL, S., HALL, P. R. & BOSE, J. L. 2016. Generation of a stable plasmid for in vitro and in vivo studies of *Staphylococcus* species. *Applied and Environmental Microbiology,* 82**,** 6859-6869.
6. PENDLETON, A., YEO, W. S., ALQAHTANI, S., DIMAGGIO, D. A., JR., STONE, C. J., LI, Z., SINGH, V. K., MONTGOMERY, C. P., BAE, T. & BRINSMADE, S. R. 2022. Regulation of the Sae Two-Component System by branched-chain fatty acids in *Staphylococcus aureus*. *mBio,* 13**,** e0147222.
7. DOS SANTOS FERREIRA, M.C., PENDLETON, A., YEO, S., MÁLAGA GADEA, F. C., CAMELO, D., MCGUIRE, M., AND BRINSMADE, S. R. (2024). In *Staphylococcus aureus*, the acyl-CoA synthetase MbcS supports branched-chain fatty acid synthesis from carboxylic acid and aldehyde precursors. *Molecular Microbiology*, *121*(5), 865-881.
8. WATERS, N. R., SAMUELS, D. J., BEHERA, R. K., LIVNY, J., RHEE, K. Y., SADYKOV, M. R. & BRINSMADE, S. R. 2016. A spectrum of CodY activities drives metabolic reorganization and virulence gene expression in *Staphylococcus aureus*. *Mol Microbiol.,* 101**,** 495-514.
9. ROCCO, C. J., DENNISON, K. L., KLENCHIN, V. A., RAYMENT, I. & ESCALANTE-SEMERENA, J. C. 2008. Construction and use of new cloning vectors for the rapid isolation of recombinant proteins from *Escherichia coli*. *Plasmid,* 59**,** 231-7.
10. MLYNEK, K. D., L. L. BULOCK, C. J. STONE, L. J. CURRAN, M. R. SADYKOV, K. W. BAYLES AND S. R. BRINSMADE (2020). Genetic and biochemical analysis of CodY-mediated cell aggregation in *Staphylococcus aureus* reveals an interaction between extracellular DNA and polysaccharide in the extracellular matrix. *J Bacteriol* 202(8).
